# Supplementary material for: A cell membrane model that reproduces cortical flow-driven cell migration and collective movement
Source: Front Cell Dev Biol. 2023 Jun 23;11:1126819. doi: 10.3389/fcell.2023.1126819 (PMC10328438; doi:10.3389/fcell.2023.1126819)
Supplement: Supplementary file 6 [file DataSheet1.pdf]

Supplementary Material

A cell membrane model that reproduces cortical flow-driven cell migration and collective movement

Katushiko Sato*

*** Correspondence:** Katsuhiko Sato: ksato@sci.u-toyama.ac.jp

This supplementary material file contains four appendices A-D, legends of supplementary movies S1-S14, and supplementary figures S1 and S2 in order.

Appendix A: Derivation of the form of W in Equation (1)

In this appendix, we derive the form of a dissipation function *W*, which expresses the frictional forces exerted on the segments by the substrate. To start, we first describe the motion of a single segment in two-dimensional (2D) space; $\mathbf{R} = (X, Y)$ indicates the center of the segment, and θ is the angle between the segment and the x-axis. Each part of the segment is specified by a scalar ξ, with a value that spans the range $[-\ell/2, \ell/2]$, where ℓ is the length of the segment ($\ell > 0$). The position of the point in the segment specified by ξ is expressed as $\mathbf{r} = (X + \chi(\xi)\cos\theta, Y + \chi(\xi)\sin\theta)$, where $\chi(\xi)$ is the counter length of the point measured from the center of the segment. We set $\chi(\xi) = \xi$ at the current time *t*, and the quantities X, Y, θ, $\chi(\xi)$, and ℓ are all functions of time *t;* that is, the segment translates, rotates, and extends (or shrinks) on the substrate. The velocity $\mathbf{v}$ of the portion of the segment specified by ξ is calculated as

$$\mathbf{v} = \frac{d\mathbf{r}}{dt} = (\dot{X} + \dot{\chi}(\xi)\cos\theta + \dot{\theta}\xi(-\sin\theta), \dot{Y} + \dot{\chi}(\xi)\sin\theta + \dot{\theta}\xi\cos\theta),$$

Equation (A.1)

where the dot over a variable indicates its time derivative and we have used $\chi(\xi) = \xi$. We further assume that extension (or shrinkage) of the segment is homogeneous within the segment; that is,

$$\dot{\chi}(\xi) = \xi\frac{\dot{\ell}}{\ell}.$$

Equation (A.2)

Next, we decompose the velocity $\mathbf{v}$ in Equation (A.1) into components that are parallel and perpendicular to the segment as

$$\begin{aligned}\mathbf{v} &= (\mathbf{v}\cdot\mathbf{e})\mathbf{e} + [\mathbf{v} - (\mathbf{v}\cdot\mathbf{e})\mathbf{e}] \\ &= \mathbf{v}_{\parallel} + \mathbf{v}_{\perp}\end{aligned},$$

Equation (A.3)

where $\mathbf{e}$ is the unit vector pointed in the direction of the segment, expressed by $\mathbf{e} = (\cos\theta, \sin\theta)$. In the main text, we have assumed that when the region of the segment specified by the range $[\xi,\ \xi + d\xi]$ moves parallel to the segment, it bears a frictional force $-\eta_{\parallel}\mathbf{v}_{\parallel}d\xi$ from the substrate, whereas when that region moves perpendicular to the segment, it bears a frictional force $-\eta_{\perp}\mathbf{v}_{\perp}d\xi$ from the substrate. The total frictional force that the whole segment bears from the substrate is expressed by the following dissipation function

$$W_{substrate} = \frac{\eta_{\parallel}}{2}\int_{-\frac{\ell}{2}}^{\frac{\ell}{2}} \mathbf{v}_{\parallel}{}^{2} d\xi + \frac{\eta_{\perp}}{2}\int_{-\frac{\ell}{2}}^{\frac{\ell}{2}} \mathbf{v}_{\perp}{}^{2} d\xi .$$

In addition, we assumed that when a segment extends or shrinks (*i.e.*, the length ℓ of the segment changes with time), an internal friction arises within the segment, which has a strength proportional to the strain rate of that extension (or shrinkage) of the segment. Because the strain rate at ξ in the segment is expressed by $\partial\dot{\chi}(\xi)/\partial\xi = \dot{\ell}/\ell$, the dissipation function for this internal frictional force is calculated as

$$W_{inner} = \frac{\mu}{2}\int_{-\frac{\ell}{2}}^{\frac{\ell}{2}} \left(\frac{\dot{\ell}}{\ell}\right)^{2} d\xi ,$$

where μ is the frictional coefficient of the internal friction. Thus, using Equation (A.3), the total dissipation function for a single segment is expressed as

$$\begin{aligned}W_{\text{single}} &= W_{substrate} + W_{inner} \\ &= \frac{\eta_{\parallel}}{2}\int_{-\frac{\ell}{2}}^{\frac{\ell}{2}} (\mathbf{v}\cdot\mathbf{e})^{2} d\xi + \frac{\eta_{\perp}}{2}\int_{-\frac{\ell}{2}}^{\frac{\ell}{2}} (\mathbf{v}^{2} - (\mathbf{v}\cdot\mathbf{e})^{2}) d\xi + \frac{\mu}{2}\int_{-\frac{\ell}{2}}^{\frac{\ell}{2}} \left(\frac{\dot{\ell}}{\ell}\right)^{2} d\xi .\end{aligned}$$

Equation (A.4)

By further using Equations (A.1) and (A.2), we can evaluate the integrals in Equation (A.4) to yield

$$\begin{aligned}W_{\text{single}} &= \frac{\eta_{\parallel}\ell}{2}\left((\dot{X}\cos\theta + \dot{Y}\sin\theta)^{2} + \frac{(\dot{\ell})^{2}}{12} \right) \\ &\quad + \frac{\eta_{\perp}\ell}{2}\left[\left(\dot{X}^{2} + \dot{Y}^{2} + \frac{(\dot{\ell})^{2}}{12} + \frac{(\dot{\theta}\ell)^{2}}{12} \right) - \left((\dot{X}\cos\theta + \dot{Y}\sin\theta)^{2} + \frac{(\dot{\ell})^{2}}{12} \right) \right] + \frac{\mu}{2}\frac{(\dot{\ell})^{2}}{\ell} .\end{aligned}$$

This expression is true even when there are many segments on the substrate. Thus, the dissipation function for frictional forces acting on the segments comprising the polygons on the substrate is expressed by

$$W = \sum_{\langle ij \rangle} \left\{ \frac{\eta_{\parallel}^{(ij)} \ell_{ij}}{2} \left((\dot{X}_{ij} \cos\theta_{ij} + \dot{Y}_{ij} \sin\theta_{ij})^2 + \frac{(\dot{\ell}_{ij})^2}{12} \right) \right.$$
$$\left. + \frac{\eta_{\perp}^{(ij)} \ell_{ij}}{2} \left[\left(\dot{X}_{ij}^{\,2} + \dot{Y}_{ij}^{\,2} + \frac{(\dot{\ell}_{ij})^2}{12} + \frac{(\dot{\theta}_{ij} \ell_{ij})^2}{12} \right) - \left((\dot{X}_{ij} \cos\theta_{ij} + \dot{Y}_{ij} \sin\theta_{ij})^2 + \frac{(\dot{\ell}_{ij})^2}{12} \right) \right] + \frac{\mu^{(ij)}}{2} \frac{(\dot{\ell}_{ij})^2}{\ell_{ij}} \right\},$$

Equation (A.5)

where the index ij indicates that the quantity is that of the segment ij, consisting of the i-th and j-th nodes. The symbol $\langle ij \rangle$ under the summation symbol indicates that the sum is taken over all the segments in the system. Equation (A.5) is in fact written by using $\mathbf{e}_{ij} = (\mathbf{r}_j - \mathbf{r}_i) / \ell_{ij}$ and $\mathbf{v}_i = \dot{\mathbf{r}}_i$ as

$$W = \sum_{\langle ij \rangle} \left\{ \frac{\eta_{\parallel}^{(ij)} \ell_{ij}}{6} \left((\mathbf{e}_{ij} \cdot \mathbf{v}_i)^2 + (\mathbf{e}_{ij} \cdot \mathbf{v}_i)(\mathbf{e}_{ij} \cdot \mathbf{v}_j) + (\mathbf{e}_{ij} \cdot \mathbf{v}_j)^2 \right) \right.$$
$$+ \frac{\eta_{\perp}^{(ij)} \ell_{ij}}{6} \left[\left((\mathbf{v}_i)^2 + (\mathbf{v}_i \cdot \mathbf{v}_j) + (\mathbf{v}_j)^2 \right) - \left((\mathbf{e}_{ij} \cdot \mathbf{v}_i)^2 + (\mathbf{e}_{ij} \cdot \mathbf{v}_i)(\mathbf{e}_{ij} \cdot \mathbf{v}_j) + (\mathbf{e}_{ij} \cdot \mathbf{v}_j)^2 \right) \right]$$
$$\left. + \frac{\mu^{(ij)}}{2} \frac{((\mathbf{r}_j - \mathbf{r}_i) \cdot (\mathbf{v}_j - \mathbf{v}_i))^2}{\ell_{ij}^{\;3}} \right\}.$$

Equation (A.6)

Because all the variables $\{X_{ij}, Y_{ij}, \theta_{ij}, \ell_{ij}\}$ contained in Equation (A.5) are written in terms of the positions of nodes $\{\mathbf{r}^{(i)}\}$, where $\mathbf{r}^{(i)}$ is the position of the i-th node, the function W is a function of $\{\mathbf{r}^{(i)}\}$ and $\{\dot{\mathbf{r}}^{(i)}\}$.

Appendix B: Shrinkage of a circular cell surface with a constant surface tension and no constraints on the area and perimeter of the cell

In this appendix, we consider a continuous circular membrane on a substrate, which bears a constant surface tension $\gamma = \gamma_0$, as well as frictional forces from the substrate and intrinsic motion of the membrane. We assume that this circular cell has no constraints on the area and perimeter of the circular membrane, which is expressed by $K = 0$, $K_p = 0$, $\kappa^{(ijk)} = 0$ in Equation (2). As a result, the constant surface tension causes the circular membrane to shrink about its origin, keeping forces balanced between the surface tension and frictional forces exerted on the membrane. In this case, the mechanical potential U of the membrane is expressed by

$$U = \gamma_0 (2\pi r),$$

Equation (B.1)

where r is the radius of the circular membrane. The dissipation function W of this circular membrane, which reflects the frictional forces exerted on the membrane, is expressed by

$$W = \frac{\eta}{2}(\dot{r})^2 (2\pi r) + \frac{\mu}{2}(\frac{\dot{r}}{r})^2 (2\pi r),$$

Equation (B.2)

where we have assumed that any part of the membrane moves only in the radial direction and used the fact that the strain rate of the membrane is equal to $\dot{r}/r$. The force balance equation for r is $-\frac{\partial U}{\partial r} - \frac{\partial W}{\partial \dot{r}} = 0$, which gives the following time evolution equation for r:

$$\gamma_0 + \eta \dot{r} r + \mu \frac{\dot{r}}{r} = 0.$$

Equation (B.3)

Solving Equation (B.3) under the initial condition $r(0) = r_0$ yields

$$r(t) = \sqrt{\frac{\mu}{\eta} W_0 (\frac{\eta}{\mu} e^{-\frac{2}{\mu}(\gamma_0 t - (\frac{\eta}{2} r_0{}^2 + \mu \log r_0))})},$$

Equation (B.4)

where $W_0(z)$ is the Lambert W function (*i.e.*, the product logarithm) that satisfies $z = W_0(z) e^{W_0(z)}$ for $z > -1/e$ and has the property $dW_0(z)/dz\,|_{z=0} = 1$. At the limit $\mu \to +0$, Equation (B.4) becomes

$$r(t) = \sqrt{\frac{2}{\eta}(\frac{\eta}{2}r_0^{\ 2} - \gamma_0 t)}\ ,$$

indicating that the circular membrane converges to its center with a finite time interval $t = \eta r_0^{\ 2} / (2\gamma_0)$, whereas when $\mu > 0$, the circular membrane does not converge to the center within a finite time.

Appendix C: Analytical calculation for the velocity of a cell moving on a substrate due to direction-dependent surface tension

In this appendix, we analytically calculate the steady-state velocity of a cell moving with a direction-dependent surface tension. For the following analyses, we fix the shape of the cell as either a circle or as two parallel lines connected at both ends by semicircles, illustrated in Figure 3C. The former shape corresponds to the simulations for the parameters listed in the caption of Figure 2A, in which the shape of the cell is circular. The latter corresponds to the simulations for the parameters specified in the caption of Figure 2D, in which the cell is sandwiched by a potential and elongates, becoming sausage-like in shape. Here, our method is basically the same as that utilized in a previous study [1]. However, unlike in the published report, the present model considers the internal friction in the cell membrane, and the cell shape is not necessarily circular.

For the circular case, consider a cell on a substrate, which consists of a surface and an inner domain. Here, we account only for the dynamics of the cell surface and do not explicitly consider any dynamics of the inner domain. The surface is represented by a closed curve on the substrate, which is assumed to be a circle, having a constant radius of R. The surface of the cell also has a surface tension, with a strength that is dependent on the relative position of the surface under consideration. This surface tension strength is denoted by $\gamma(\theta)$, where θ is the angle between the x-axis and the vector connecting the center of the cell and the surface under consideration. We assume that the cell is polarized to the x-direction, which is described by the form of $\gamma(\theta)$. Due to the symmetry about the x-axis, γ satisfies $\gamma(\theta) = \gamma(-\theta)$. The frictional forces exerted on the surface are the same as those for the membrane model described by Equation (1) in the main text. That is, if parts of the cell surface move with respect to the substrate, they bear a frictional force from the substrate. If there is relative movement within the surface (*e.g.*, extension or contraction of the surface), that movement creates internal friction within the surface. For this surface movement, we do not consider any inertia, such that, at each part of a surface, the forces created by surface tension and friction are balanced at all times.

With these parameters established, we then calculate the steady-state velocity of this circular cell, as follows. We first discretize the circle by splitting it into N arcs and N nodes to describe the dynamics of the surface, where N is a large positive integer that ultimately goes to infinity at the last step. The direction of cell movement must also be in the x-direction due to the symmetry of the system. Thus, without loss of generality, we can express the position $\mathbf{r}_i$ of the i-th node as

$$\mathbf{r}_i = (X + R\cos\theta_i, R\sin\theta_i),$$

Equation (C.1)

where X is the x-component of the position of the cell center, and θ_i is the angle between the x-axis and the vector connecting the center of the cell and the i-th node. The index i runs from 0 to N-1, and X and θ_i are time-dependent variables of this system. We set the initial conditions for X and θ_i as

$$\theta_i(0)=\frac{2\pi}{N}i \qquad \text{for } i=0,1,2,...,N-1 .$$
$$X(0)=0$$

Equation (C.2)

Using Equation (C.1), we can write the potential function U and dissipation function W of this system as

$$U=\sum_{i=0}^{N-1}(\theta_{i+1}-\theta_i)\gamma_{ii+1}R\text{, and}$$

Equation (C.3)

$$W=\frac{\eta}{2}(\frac{2\pi R}{N})\sum_{i=0}^{N-1}\left[(\dot{X}-R\dot{\theta}_i\sin\theta_i)^2+(R\dot{\theta}_i\cos\theta_i)^2\right]+\frac{\mu}{2}\sum_{i=0}^{N-1}\left[\frac{R(\dot{\theta}_{i+1}-\dot{\theta}_i)^2}{(2\pi/N)}\right],$$

Equation (C.4)

respectively, where γ_{ii+1} is the surface tension on the arc sandwiched by the i-th and ($i+1$)-th nodes, η is the coefficient of friction between the substrate and the surface of the cell, and μ is the coefficient of the internal friction coming from the relative movement of the parts of the surface. In Equations (C.3) and (C.4), $\theta_N(t)=\theta_0(t)+2\pi$, and the dot over a quantity indicates its time derivative. The force balance at the i-th node, expressed by $\frac{1}{(2\pi R/N)}(-\frac{\partial U}{\partial\theta_i}-\frac{\partial W}{\partial\dot{\theta}_i})=0$, yields

$$(-\gamma_{ii+1}+\gamma_{i-1i})R+\eta(\frac{2\pi R}{N})R\left[-\dot{X}\sin\theta_i+R\dot{\theta}_i\right]+\frac{\mu R}{(2\pi/N)}\left[2\dot{\theta}_i-\dot{\theta}_{i+1}-\dot{\theta}_{i-1}\right]=0,$$

Equation (C.5)

and the force balance about X, expressed by $-\frac{\partial U}{\partial X}-\frac{\partial W}{\partial\dot{X}}=0$, yields

$$\eta(\frac{2\pi R}{N})\sum_{i=0}^{N-1}\left[(\dot{X}-R\dot{\theta}_i\sin\theta_i)\right]=0.$$

Equation (C.6)

Before taking the continuous limit of these equations for X and θ_i, we rewrite θ_i as

$$\theta_i(t)=\theta(\xi_i,t)$$

Equation (C.7)

by introducing a new index ξ_i, as

$$\xi_i = \frac{2\pi}{N} i \text{ (i=0, 1, 2, …, N-1).}$$

Equation (C.7.1)

This index ξ_i plays a role in the material coordinates assigned to the cell surface. By using Equation (C.7), we can rewrite Equations (C.5) and (C.6) as

$$-\frac{\gamma(\theta(\xi_{i+1},t)) - \gamma(\theta(\xi_i,t))}{\Delta\xi} + \eta R\left[-\dot{X}\sin\theta(\xi_i,t) + R\dot{\theta}(\xi_i,t)\right] - \mu\frac{\dot{\theta}(\xi_{i+1},t) + \dot{\theta}(\xi_{i-1},t) - 2\dot{\theta}(\xi_i,t)}{(\Delta\xi)^2} = 0$$

$$\sum_{i=1}^{N}\left[(\dot{X} - R\dot{\theta}(\xi_i,t)\sin\theta(\xi_i,t))\right]\Delta\xi = 0 ,$$

Equation (C.8)

where $\Delta\xi = \frac{2\pi}{N}$, and we set $\gamma_{ii+1} = \gamma(\theta(\xi_{i+1},t))$. Taking the limit of $N \to \infty$ in Equation (C.8) yields

$$-\frac{\partial\gamma(\theta(\xi,t))}{\partial\xi} + \eta R\left[-\dot{X}\sin\theta(\xi,t) + R\dot{\theta}(\xi,t)\right] - \mu\frac{\partial^2\dot{\theta}(\xi,t)}{\partial\xi^2} = 0$$

Equation (C.9)

and

$$\int_0^{2\pi}\left[\dot{X} - R\dot{\theta}(\xi,t)\sin\theta(\xi,t)\right]d\xi = 0 .$$

Equation (C.10)

Equation (C.9) is rewritten as

$$(1 - \frac{\mu}{\eta R^2}\frac{\partial^2}{\partial\xi^2})\dot{\theta}(\xi,t) = \frac{1}{R}(\frac{1}{\eta R}\frac{\partial\gamma(\theta(\xi,t))}{\partial\xi} + \dot{X}\sin\theta(\xi,t)),$$

Equation (C.11)

and this can be considered to be an inhomogeneous linear differential equation for $\dot{\theta}(\xi,t)$ if we regard the right-hand side of Equation (C.11) as a given function. Thus, we formally solve Equation (C.11) as

$$\dot{\theta}(\xi,t) = \int_0^{2\pi} K(\xi,\xi')\frac{1}{R}(\frac{1}{\eta R}\frac{\partial\gamma(\theta(\xi',t))}{\partial\xi'} + \dot{X}\sin\theta(\xi',t))d\xi',$$

Equation (C.12)

where K is a Green function that satisfies $(1-\frac{\mu}{\eta R^2}\frac{\partial^2}{\partial \xi^2})K(\xi,\xi') = \delta(\xi-\xi')$, with the boundary conditions $K(0,\xi') = K(2\pi,\xi')$ and $\partial K(\xi,\xi')/\partial\xi|_{\xi=0} = \partial K(\xi,\xi')/\partial\xi|_{\xi=2\pi}$. Here, $\delta(x)$ is the delta function, and the explicit form of K is $K(\xi,\xi') = \frac{1}{2\alpha(-1+e^{2\pi/\alpha})}e^{\frac{|\xi-\xi'|}{\alpha}} + \frac{1}{2\alpha(1-e^{-2\pi/\alpha})}e^{-\frac{|\xi-\xi'|}{\alpha}}$, with $\alpha = \frac{1}{R}\sqrt{\frac{\mu}{\eta}}$. Inserting Equation (C.12) into Equation (C.10) yields

$$2\pi\dot{X} - \int_0^{2\pi} d\xi \int_0^{2\pi} d\xi' K(\xi,\xi')(\frac{1}{\eta R}\frac{\partial\gamma(\theta(\xi',t))}{\partial\xi'} + \dot{X}\sin\theta(\xi',t))\sin\theta(\xi,t) = 0.$$

Equation (C.13)

Because we do not consider any inertia in this system, we only have to consider the state at *t*=0 to obtain the steady-state velocity of the cell. Hence, we set *t*=0 in Equation (C.13) to yield

$$\dot{X}(0) = \frac{1}{\pi\eta R}\frac{1+(\mu/\eta R^2)}{1+(2\mu/\eta R^2)}\int_0^{2\pi} d\xi \int_0^{2\pi} d\xi' K(\xi,\xi')(\frac{\partial\gamma(\xi')}{\partial\xi'})\sin\xi,$$

Equation (C.14)

where we have used $\theta(\xi,0) = \xi$, which comes from Equations (C.2) and (C.7.1).

If we put $\gamma(\theta) = \gamma_0 - a\cos\theta$ with a constant a, which represents the degree of cell polarization, into Equation (14), we obtain

$$\dot{X}(0) = \frac{a}{\eta R}\frac{1}{1+2(\mu/\eta R^2)},$$

Equation (C.15)

which is the analytical form of the velocity for a circular cell. Importantly, this result coincides with numerical simulations of the membrane model described in Equations (1)–(3), for $K=100$, $K_p = 0$, $\kappa^{(ijk)} = 0$, and $A_0 = \pi$ (see Figure 2B, C).

The solutions for the case of $\mu = 0$ and $\gamma(\theta) = \gamma_0 - a\cos\theta$ are useful for checking various relations that hold in circular cell migration, with relevant quantities for that case summarized as follows. The position $\mathbf{r}$ and velocity $\mathbf{v}$ of the cell surface point specified by θ are

$$\mathbf{r}(\theta) = (\frac{a}{\eta R}t + R\cos\theta, R\sin\theta)$$
$$\mathbf{v}(\theta) = (\frac{a}{\eta R} - \frac{2a}{\eta R}(\sin\theta)^2, \frac{2a}{\eta R}\sin\theta\cos\theta),$$

Equation (C.16)

respectively, where we have used $\dot{X}(0) = \frac{a}{\eta R}$ and $\dot{\theta} = \frac{2a}{\eta R^2}\sin\theta$, which are obtained from Equations (C.12) and (C.15) and $K(\xi,\xi') = \delta(\xi - \xi')$.

The above method is also used for obtaining the steady-state velocity of a cell whose shape is not circular. For example, if a cell is sandwiched by two walls, the shape of that cell becomes elongated, as shown in Figure 3A. When $\mu = 0$, we can analytically calculate the steady-state velocity as

$$\dot{X}(0) = \frac{2}{\pi\eta d}\left(\int_0^{\pi/2}\sin\xi_1 \frac{d\gamma(\theta_{forward}(\xi_1))}{d\xi_1}d\xi_1 + \int_{\pi/2}^{\pi}\sin\xi_2 \frac{d\gamma(\theta_{backword}(\xi_2))}{d\xi_2}d\xi_2\right) + \frac{2\Delta\gamma}{\pi\eta d},$$

Equation (C.17)

where $\theta_{forward}(\xi_1) = \arctan(\frac{d\sin\xi_1}{d\cos\xi_1 + L_x})$, $\theta_{backword}(\xi_2) = \arctan(\frac{d\sin\xi_2}{d\cos\xi_2 - L_x})$, and $\Delta\gamma = \gamma(\pi - \theta_1) - \gamma(\theta_1) = 2aL_x / \sqrt{L_x^2 + d^2}$. The angles $\theta_{forward}$, $\theta_{backward}$, ξ_1, and ξ_2 used here are defined as shown in Figure 3C. The lengths L_x and d are the half-length of the straight part of the cell and the radius of the circular part of the cell, respectively. If the total area of the cell is constrained at $A = A_0$, L_x and d are related as $4L_xR + \pi d^2 = A_0$. The speed of the cell given by Equation (C.17) is in good agreement with the results of numerical simulations for a cell sandwiched by two parallel walls (Figure 3B).

Appendix D: Derivation of Equation (9)

In our model, the density ρ of a cell surface is defined as the amount of cell surface components per unit length. We further assume that cell surface components are allowed to flow into or out of the cell surface from the inside of the cell. By considering the amount of cell surface components in the region between the material coordinate ξ and $\xi+\Delta\xi$, as well as the material balance between time t and $t+\Delta t$, we obtain

$$\rho(\xi,t+\Delta t)\,|\,\mathbf{r}(\xi+\Delta\xi,t+\Delta t)-\mathbf{r}(\xi,t+\Delta t)\,|= \\ \rho(\xi,t)\,|\,\mathbf{r}(\xi+\Delta\xi,t)-\mathbf{r}(\xi,t)\,|+J(\xi,t)\,|\,\mathbf{r}(\xi+\Delta\xi,t)-\mathbf{r}(\xi,t)\,|\,\Delta t\text{'}$$

Equation (D.1)

where $\mathbf{r}(\xi,t)$ is the position vector of the cell surface at ξ and t, and $J(\xi,t)$ is the flux of cell surface components from the cell inside to the cell surface at ξ and t per unit length. Using the relation $\mathbf{r}(\xi,t+\Delta t)=\mathbf{r}(\xi,t)+\mathbf{v}(\xi,t)\Delta t$, where $\mathbf{v}(\xi,t)$ is the velocity of the cell surface at ξ and t, which is true to the first order in Δt, we can rewrite Equation (D.1) to the first order in Δt as

$$\rho(\xi,t+\Delta t)(1+(\Delta\mathbf{r}\cdot\Delta\mathbf{v}/(\Delta s)^2)\Delta t)=\rho(\xi,t)+J(\xi,t)\Delta t,$$

Equation (D.2)

where $\Delta\mathbf{r}=\mathbf{r}(\xi+\Delta\xi,t)-\mathbf{r}(\xi,t)$, $\Delta\mathbf{v}=\mathbf{v}(\xi+\Delta\xi,t)-\mathbf{v}(\xi,t)$ and $\Delta s=|\Delta\mathbf{r}|$. Taking the limits of $\Delta\xi\to+0$ and $\Delta t\to+0$, we obtain

$$\frac{\partial\rho(\xi,t)}{\partial t}=-\frac{(\partial\mathbf{r}/\partial\xi)\cdot(\partial\mathbf{v}/\partial\xi)}{(\partial s/\partial\xi)^2}\rho(\xi,t)+J(\xi,t),$$

Equation (D.3)

where $s(\xi,t)$ is the counter length of the surface point at ξ from some reference point on the cell surface. Equation (D.3) is Equation (9) in the main text.

References

1. Okuda S, Sato K. Polarized interfacial tension induces collective migration of cells, as a cluster, in a 3D tissue. *Biophys J.* (2022) Apr 28:S0006-3495(22)00317-4. doi: 10.1016/j.bpj.2022.04.018

Legends of Supplementary movies S1-S14

Supplementary Movie S1: A circular cell migrates in the x-direction due to the direction-dependent surface tension in Equation (4). The parameter values used here are indicated in the legend of Figure 2A.

Supplementary Movie S2: A non-circular cell migrates due to the direction-dependent surface tension in Equation (4). The parameter values used here are indicated in the legend of Figure 2D.

Supplementary Movies S3-S10: Cells in a cluster migrate due to direction-dependent surface tension. The common parameters used in these simulations are indicated in the legend of Figure 4B. The individual parameters are: Movie S3 ($N_{cell} = 2$, $\gamma_b = 0.1$), Movie S4 ($N_{cell} = 2$, $\gamma_b = 0.9$), Movie S5 ($N_{cell} = 3$, $\gamma_b = 0.1$), Movie S6 ($N_{cell} = 3$, $\gamma_b = 0.9$), Movie S7 ($N_{cell} = 4$, $\gamma_b = 0.1$), Movie S8 ($N_{cell} = 4$, $\gamma_b = 0.9$), Movie S9 ($N_{cell} = 10$, $\gamma_b = 0.1$), and Movie S10 ($N_{cell} = 10$, $\gamma_b = 0.9$).

Supplementary Movies S11 and S12: Cells in a cluster rotate when the polarity of each cell is tilted with respect to the cluster center. The parameters used here are indicated in the legend of Figure 5B. Movie S11: $\gamma_b = 0.2$, Movie S12: $\gamma_b = 1.0$.

Supplementary Movies S13 and S14: Cell movement for the case where the direction of cell polarity changes with time, according to the velocity alignment mechanism given in the existing works [41], in which the direction of polarity of cell α obey the equation $\dot{\theta}_p^{(\alpha)} = -\frac{1}{\tau_0}\sin(\theta_p^{(\alpha)} - \theta_{velocity}^{(\alpha)})$, where $\theta_p^{(\alpha)}$ is the angle between the direction of polarity of cell α and the *x* direction and $\theta_{velocity}^{(\alpha)}$ is the angle between the direction of velocity of cell α and the *x* direction. τ_0 is a relaxation time. We have put $\tau_0 = 1.0$ and $\gamma_b = 0.2$. The other parameters are the same as those in the legend of Figure 5B. The initial $\theta_p^{(\alpha)}$ is given by a random number uniformly distributed in $[0, 2\pi]$.The red arrows in the movies indicate the directions of polarity of cells. Movies S13 and S14 start with different initial values of $\{\theta_p^{(\alpha)}\}$.

Supplementary Figures

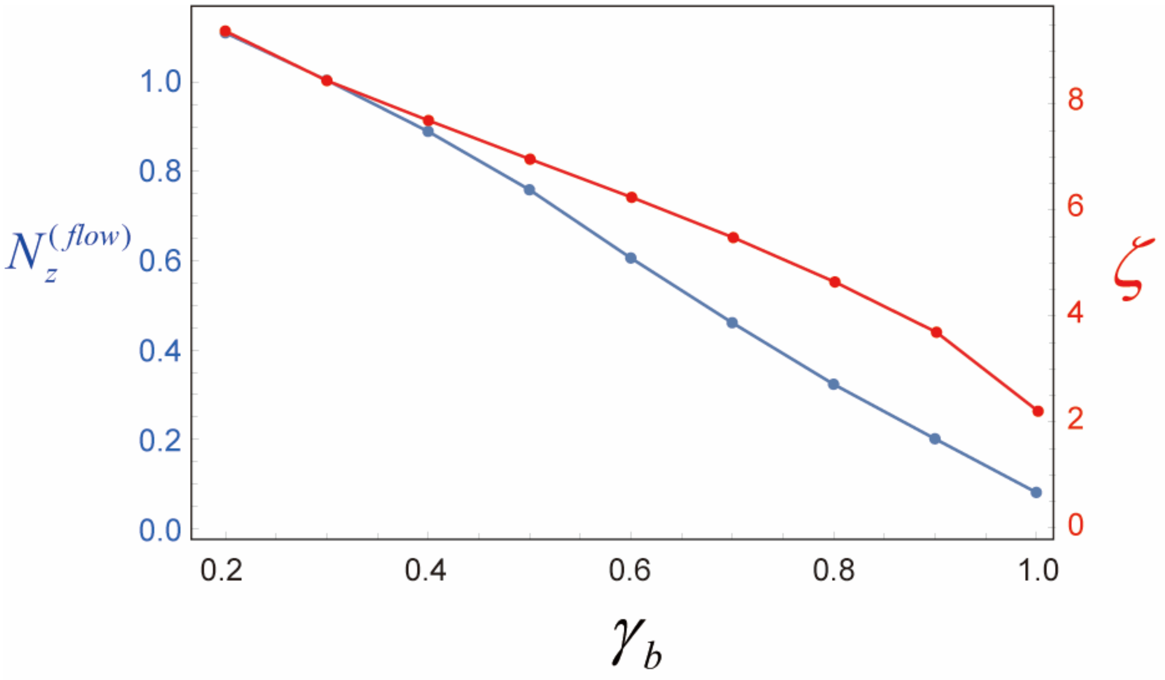

Supplementary Figure S1. Plots of $N_z^{(flow)}$ (blue) and ζ (red), which are defined in section 3.6 in the main text, as functions of γ_b. We see that the decrease in ζ with increased γ_b is a little gentler than that in $N_z^{(flow)}$, which leads to a decrease in $\omega = N_z^{(flow)} / \zeta$ with increased γ_b (Figure 5C).

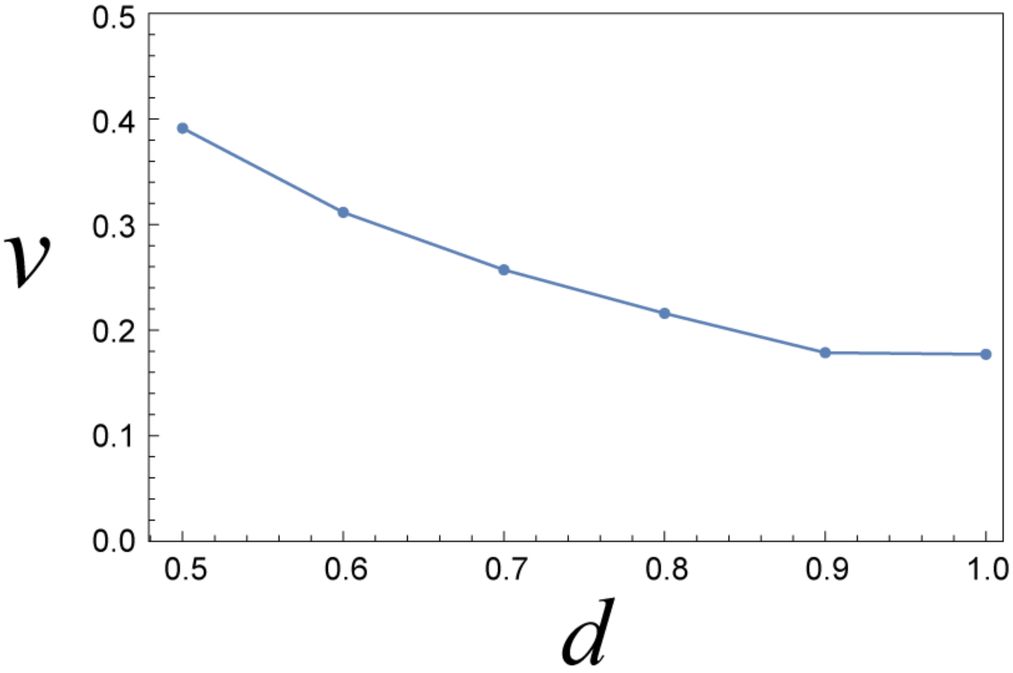

Supplementary Figure S2. The steady speed, v, of a cell sandwiched by walls as a function of the distance between two walls, d. In these simulations, the the value $\hat{\gamma}_{ij}$ assigned to the cell boundary is constantly increased along the cell surface from the front to the rear of the cell, which setup is different from Equation (4). The maximum and minimum values of $\hat{\gamma}_{ij}$ are the same as in the simulations in Fig. 3, i.e., $\hat{\gamma}_{ij} = 1.2$ at the rear and $\hat{\gamma}_{ij} = 0.8$ at the front. As in Fig. 3B, the steady speed v is increased with the decrease in d. The depencend v on d is relatively moderate compared with Fig. 3B.
